# Items, Selections and Remarks

**Published:** 1870-12

**Authors:** W. W. Miner


### Items, Selections and Remarks.

BY W. W. MINER.

An apparatus called "the gastric douche," and similar in design to a nasal douche, has been suggested by Dr. Ploss, of Leipsic. It is thought that it may be useful in cases of poisoning, and in diseased conditions of the stomach.—Dr. W. H. Gobrecht, of Cincinnati, in the Transactions of the Pennsylvania State Medical Society, reports a case of poisoning, where a young man, aged about twenty years, had taken seven-eights of an ounce of a saturated solution of strychnia, with crystals in excess, in chloroform, both by inhalation and ingestion, without any other result than complete and prolonged anæsthetization, and some temporary subsequent numbness, with no known injurious consequences after two months had elapsed.—*Med Record.*—Prof. Moses Greene, of Rush Medical College, states in the Chicago Medical Journal that he has found in his practice that skating is a fertile source of necrosis of the tibia in young men and boys.—Cocoa is recommended as an excellent agent for disguising the taste of quinine.—M. Didierjean, in a communication to the Academy of Sciences, states that, in his manufactory, workmen suffered more or less from lead poisoning, in spite of the use of sulphuric acid, lemonade, baths, open air work, &c. He says further, that three years since, his attention was called to the fact that certain of his workmen who constantly used milk at their daily meals, were unaffected by lead poisoning, and he accordingly recommended the use of milk to the other workmen, and for the last eighteen months he says not a single workman has been affected with lead poisoning.—Chloride of Aluminium has been recommended as an antiseptic and deodorizer of great utility, by Mr. Squire, of England, who has also used this remedy in the form of spray, as an application to the throat in diphtheria.—Dr, J. F. Snyder, of Virginia, Cass Co., Illinois, reports in the Chicago Medical Examiner the case of a man who was "stabbed in the back, at the lower point of the shoulder blade," but as the wound soon healed, no farther cause of trouble was suspected. Twelve years after, and when the patient was sixty years of age, in a paroxysm of coughing, he threw up from the right bronchus an ounce or two of pus, and the point of a knife as it proved, which was an inch in length, half an inch in width, and weighed half a drachm. The pains which were experienced, more or less frequently during the twelve years in which the piece of steel was migrating from the scapula into the bronchus, were ascribed to rheumatic origin.—A writer in the British Medical Journal, says that dogs which have been poisoned by strychnine, have apparently had their lives saved by being administered doses of oil, and by having been hung up for some time by the hind legs with the head downwards. As to the modus operandi of this treatment, he says that the oil may mechanically obstruct the absorption of the poison by the stomach, while the cerebral congestion which is occasioned, may counteract in some way the effects of the poison on the nervous system. He accordingly suggests that the inclination of the head downwards in cases of poisoning by strychnia in man may be of utility.—Prof. Von Graefe's annual income from his professional duties is stated to have been one hundred thousand dollars, while that from his paternal estate was twenty-five thousand dollars.—The Library of the College of Physicians and Surgeons of New York City contains fifteen thousand volumes.—A case is given in the British Medical Journal of a man who had been run over by a railway train, upon whose body there was not the smallest wound, and only a few abrasions of cuticle across the abdomen, but, on opening the abdomen, all the abdominal muscles were found to be cut through, as also the right kidney, the transverse colon and ileum, while the third lumbar vertebra was crushed literally to powder.—Joseph G. Richardson, M. D., author of the recent work on "Medical Microscopy," publishes in the Baltimore Medical Journal, an account of his experiments demonstrating the identity of the white corpuscles of the blood, with the salivary, pus and mucous corpuscles.—It is reported that a son of the surgeon Langenbech has died of wounds received in the Franco-Prussian war, and that sons of Drs. Simon, Stilling, and Lauer have been wounded. —Messieurs Legros, Onimus and Cyon have recently been awarded valuable medals for their researches on the application of electricity to therapeutics.—The prize of one hundred dollars in gold, offered by the American Journal of Obstetrics, for the best essay on the morbid anatomy of the placenta, has been awarded to Jas. P. Whittaker, M. D., of Cincinnati.—Henry J. Bigelow, M. D., of Boston, has presented the Massachusetts General Hospital a specially ordered set of surgical instruments, with a fund adequate to their renewal, also, money for the support of a free bed in the institution for five years.—The Library of the late Professor Von Graefe, is in the hands of Hirschwald, one of the Berlin booksellers.—Professor Willard Parker has resigned his chair of surgery at the College of Physicians and Surgeons, and is succeeded by Prof. Markoe.—It is said in the *Lancet*, for November, that the students of the University of Edinburgh manifested their disapproval of the appointment of the son of the late Professor Simpson to his father's position by riotous and unmannerly conduct during the first appearance of the new Professor in his lecture-room.—Dr. J. Gibbons Hunt, of Philadelphia, states that the amœboid movements observed in the white blood corpuscles are not peculiar to animal life, but are even more actively performed in the nucleus of the cell of anacharis alsinastrum.—*Richmond Med. Journ.*—Dr. H. Raphael, of New York City, in a paper read before the Medico-Legal Society of that city, made mention of a method recently discovered for the determining of whether a child was born dead or alive.—It is said that collections of crystallized uric acid may be found in the kidney of a child which was born alive and died soon after birth, and that the detection of such collections furnishes as positive proof of life as does the dilatation of the lungs by air. Dr. Raphael is led to believe from cases he has lately examined, that it is superior in importance as a proof to that from the inflation of the lungs.

Wm. Wood & Co. announce that they are about to publish a work on Palsies and kindred disorders of the Nervous System, by Meredith Clymer, M. D.; a manual on Surgery, by Thomas Bryant, F. R. C. S.; a treatise on Post-Mortem Examinations, by Francis Delafield, M. D ; a revised edition of Bartholow on Spermatorrhœa; a small work on the Mortality of Childbed, by J. Matthew Duncan, M. D., etc.; also, by the same author, his work on Fecundity, Fertility and Sterility; a work on Epilepsy, by M. Gonzales Escheverria, M. D.; a practical treatise on Diseases of Women, by Robert Barnes, M. D.; a work on the Medical and General Uses of Electricity, by Drs. Beard and Rockwell; a treatise on Surgery, by Frank H. Hamilton, M. D.; a work on Albuminuria, by W. W. Dickinson, M. D.; on Diseases of the Throat, Larynx, etc., by J. Solis Cohen, M. D.; Clinical Lectures on Diseases of the Genito-Urinary Organs, by J. W. S. Gouley, M. D.; on Diseases of the Ear, by D. B. St. John Roosa, M. D.; on Obstetrics, by W. H. Byford, M. D.; Insanity and its Treatment, by G. Fielding Blandford, M. D.

